# Estimating Chinese bilateral aid for health: an analysis of AidData’s Global Chinese Official Finance Dataset Version 2.0

**DOI:** 10.1136/bmjgh-2022-010408

**Published:** 2022-12-02

**Authors:** Kaci Kennedy McDade, Paige Kleidermacher, Gavin Yamey, Wenhui Mao

**Affiliations:** Center for Policy Impact in Global Health, Duke Global Health Institute, Durham, North Carolina, USA

**Keywords:** health policies and all other topics, health economics, health policy

## Abstract

**Background:**

Although it is difficult to quantify, previous estimates suggested that China’s global health aid has increased sharply since the early 2000s. Unlike many donors, China has no official aid reporting obligations, nor does it voluntarily disclose detailed aid information. Our study aimed to create a standardised estimate using commonly accepted definitions of aid and frameworks for categorising health projects.

**Methods:**

We categorised AidData’s Chinese Official Finance Dataset health-related projects according to health aid frameworks from the Organisation for Economic Co-operation and Development (OECD) and the Institute for Health Metrics and Evaluation (IHME). Only projects that complied with the definition of official development assistance were included. We analysed the project count and financial value to assess China’s priority health aid areas.

**Findings:**

Between 2000 and 2017, China funded 1339 health-related aid projects, or 13% of its total aid project portfolio. Most of these projects were located in sub-Saharan Africa. According to the OECD framework, the priority focus areas of these projects were: medical services, such as specialty equipment and tertiary services (n=489, 37%); basic health care, such as basic medical services and drugs (n=251, 19%); malaria control (n=234, 18%) and basic health infrastructure (n=178, 13%). Under the IHME framework, health systems strengthening accounted for 74% (n=991) of total projects, primarily due to China’s contributions to human resources for health, infrastructure and equipment. The only other major allocation under the IHME framework was malaria (n=234, 18%). When we estimated missing financial values under the OECD framework, China was the fifth largest health aid donor to African countries from 2002 to 2017, after the USA, the UK, Canada and Germany.

**Conclusion:**

Our findings enable a better understanding of Chinese health aid in the absence of transparent aid reporting, which could contribute to better coordination, collaboration and resource allocation for both donor and recipient countries.

**WHAT IS ALREADY KNOWN ON THIS TOPIC**

- While the Organisation for Economic Cooperation and Development’s (OECD’s) Creditor Reporting System (CRS) is the main platform used by donor countries to track aid flows in a systematic way, some key development funders, such as China, are not included. The figures on aid presented in the CRS database underestimate total development assistance contributions.
- Although it is difficult to quantify, previous estimates suggested that China’s global health aid has increased sharply since the early 2000s. China has no official aid reporting obligations, nor does it voluntarily disclose detailed aid information, so there is uncertainty in the estimates of Chinese aid.
- Several third parties have attempted to estimate China’s health aid footprint. Unfortunately, current estimates use varied definitions of health aid, geographic regions and time spans. These distinct and differing methodological approaches make it difficult to compare Chinese aid with aid from other donors.

**WHAT THIS STUDY ADDS**

- We used commonly accepted definitions of aid and two frameworks—the OECD and the Institute for Health Metrics and Evaluation (IHME) frameworks—for categorising health projects by focus area.
- Our estimate of Chinese global health aid disaggregated by health sector focus area is comparable to health aid from other donors.
- We also use different approaches to estimate missing financial values to understand the total contribution of Chinese global health aid.

**HOW THIS STUDY MIGHT AFFECT RESEARCH, PRACTICE OR POLICY**

- Chinese health aid generally increased from 2000 to 2017, with some fluctuations, and most Chinese health aid projects were in sub-Saharan Africa.
- There are clear areas of focus for China’s health aid portfolio. According to the OECD framework, the priority areas were medical services, basic health care, malaria control and basic health infrastructure. According to the IHME framework, health systems strengthening and malaria were the priority areas.
- When we estimate missing financial values using OECD and IHME frameworks, China was respectively the fifth and sixth largest health aid donor to African countries from 2002 to 2017.
- Our findings enable a better understanding of Chinese health aid and better coordination, collaboration, and resource allocation for both donor and recipient countries.

## Introduction

Foreign aid, or official development assistance (ODA), has historically come from wealthy Western nations that are part of the Organisation for Economic Cooperation and Development’s (OECD’s) Developmental Assistance Committee (DAC). Established in 1961, the OECD DAC sets the guidelines for what is, and is not, considered ODA.^1^ Members of the DAC commit to reporting their aid statistics in a standardised way via the Creditor Reporting System (CRS). In addition to the OECD platform, other platforms for aid reporting have emerged in recent years, such as the International Aid Transparency Initiative. However, the OECD remains the primary platform used by donor countries to track aid flows in a systematic way.

While the OECD CRS is the best reflection of aid flows that is available, it has a major gap: it tracks aid flows only from DAC donors. Flows from donors outside of DAC (the ‘non-members’) are not formally tracked. Although many non-members voluntarily report their aid statistics to the DAC, some key development funders, such as China, do not. Therefore, the figures presented in the CRS database underestimate total development assistance contributions.

Given China’s large economy and expanding interest in global cooperation, it has the capacity to transform the landscape of global health aid. China has intermittently published aggregate foreign aid flows in three white papers, in 2011, 2014 and 2021. Although the most recent white paper has considerably more detail and information than previous white papers, all three papers lack project-specific information and include data that are not comparable to standardised methods of tracking aid.^2–4^ Not surprisingly, in 2020 China scored a 1.2/100 on the Aid Transparency Index, the lowest transparency score of any donor.^5^ Because of such data gaps, combined with the realisation of the increasingly important role China plays in financing development, several third parties have attempted to track and/or estimate Chinese aid. In particular, several estimates have tried to capture China’s global health aid footprint.

While existing estimation efforts are very useful for gaining insight into China’s health aid portfolio, the varied methodological approaches taken by different scholars can lead to very different results, making apples to apples comparisons with other donors challenging. In a 2020 analysis, McDade and Mao identified several key differences across five Chinese health aid estimates.^6^ Importantly, they noted that each estimate used a different definition of ‘health aid’. The scope of what is or is not considered health aid can either overinflate or underestimate China’s contributions. Several studies that McDade and Mao reviewed did not adhere to commonly used definitions of aid nor did the studies align their estimates with accepted reporting standards (eg, OECD CRS aid activity reporting framework). Additionally, McDade and Mao noted that existing studies vary substantially in terms of geographies covered, time spans included and underlying data sources.^6^ China’s global health engagement is often referred to as ‘distinctive’,^7^ and apples to apples comparison of its portfolio would enable clearer examination of why that is or is not the case.

This study aimed to address these limitations, building on previous tracking efforts in order to advance the field of estimation of China’s health aid in several ways. We provided an estimate of Chinese global health aid disaggregated by health sector focus areas in a way that is comparable to health aid from other donors. To do this, we adhered to accepted definitions of aid and we applied two commonly accepted health aid classification frameworks to categorise health aid projects by focus area: the OECD and the Institute for Health Metrics and Evaluation (IHME) frameworks. These two frameworks track aid through categorisation systems that break down the specific focus of aid projects. We also used different approaches to estimate financial values for projects with missing financial information to understand the total contribution of Chinese global health aid.

## Methods

This study expanded on the current understanding of China’s health aid portfolio. We analysed project-level data using methods consistent with accepted health aid standards and norms. We provided a financial estimate for China’s health aid portfolio (2000–2017) based on our standardised methods for counting and categorising health aid. Below, we included our data sources, inclusion and exclusion criteria, the frameworks used to categorise health aid, our coding process, estimation methods and methods for comparing China’s aid with aid from other donors.

Throughout the paper, we refer to ‘projects’, since this is the language used by AidData. For the purposes of this paper, this term is equivalent to the OECD’s ‘aid activity’.^8^

### Data sources

This study used AidData’s Global Chinese Official Finance Dataset (2000–2017, V.2.0) to analyse health-related aid projects. AidData, a research group at the College of William & Mary, Williamsburg, Virginia, USA, uses the Tracking Under-reported Financial Flows (TUFF) method to identify officially funded Chinese development projects.^9^ The TUFF methodology identifies projects for its database using four sources: (1) English and Chinese language news reports; (2) documents from Chinese ministries, embassies and economic and commercial counsellor offices; (3) aid and debt information management systems of finance and planning ministries in counterpart countries and (4) case study and field research undertaken by scholars and non-governmental organisations.^10^ AidData then triangulates identified data for consistency and performs a quality control process to score the quality of sources underlying each project and to prevent double counting of linked projects.^9^ Across the whole dataset, the average source quality score was 4.2, with 1 being the lowest (ie, a smaller number of unofficial sources) and 5 being the highest (ie, two or more official sources).^9^ Among the projects included in our analysis, the average source quality score was 4.3.

#### Inclusion and exclusion criteria

Our inclusion criteria for projects in AidData’s database were as follows:

1. Health aid projects are those labelled with sector codes ‘health’ (120) or ‘population policies/programmes and reproductive health’ (130). We used this measure of health aid since it is standard practice among researchers for tracking health aid in the OECD CRS, as outlined by Grépin *et al*.^11^ Health aid does not include allied sectors that may still have an impact on health, such as water, sanitation and hygiene.
2. Only projects that are labelled ‘recommended for aggregates’ within AidData’s dataset were included. AidData uses this measure to mark projects that have either been completed, are in the implementation phase or are pipeline commitments (ie, a firm commitment in writing with proof of backed funds).^10^ While AidData’s approach is not the exact same as a disbursement, using projects that meet this criterion can be considered a proxy for aid disbursements. We use the commitment year as the year for our time trends since disbursement data are not available. The ‘recommended for aggregates’ label also ensures that projects that are linked together are not inadvertently double counted.^10^
3. Only projects that meet the requirements for ODA (referred to as ODA-like) are reported, unless otherwise noted. AidData used the OECD criteria for classifying flows to ensure its definition of aid is consistent with commonly accepted standards.

**Figure 1:**
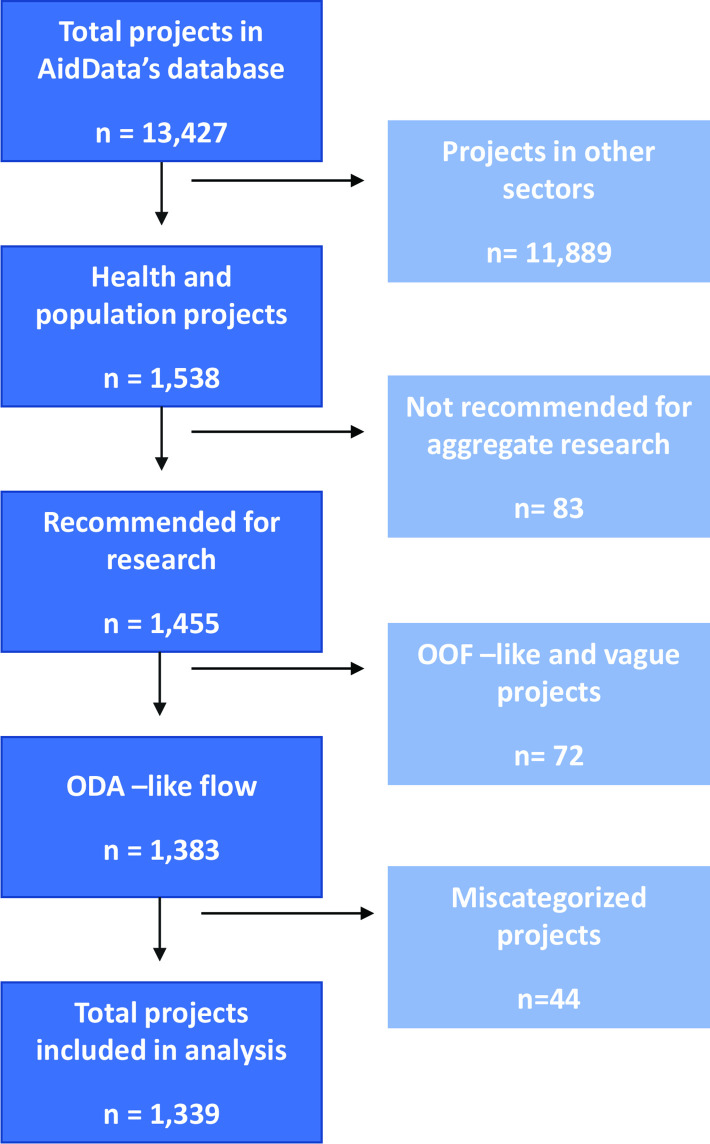
Projects included in analysis. ODA, official development assistance. OOF, other official flows.

Figure 1 is a flow chart that illustrates the number of projects screened, included and excluded for analysis according to the above criteria. We reviewed all projects that met the criteria for inclusion for accuracy prior to conducting our analysis. Specifically, we sought to identify any projects that may have been miscategorised as a health aid project. The most common misclassification we found was for projects that were humanitarian in nature but had a health component, and therefore were included as a health project. Through this quality control process, we identified 44 projects that we believed to be miscategorised and therefore we excluded these 44 from our coding exercise and analysis. We have listed project identifications (IDs), titles and our rationale for exclusion for these projects in online supplemental annex 1. Throughout this paper, we use the term health aid to represent ODA-like projects encompassing both sector codes 120 and 130, unless otherwise noted.

10.1136/bmjgh-2022-010408.supp1 Supplementary data

### Disaggregating health subsectors

AidData aims to align its database with OECD CRS standards, which enables more meaningful comparison across donors. However, one limitation of the AidData database is that it only codes projects at the sector level (eg, health, education, etc), rather than the more detailed subsector level (eg, malaria control, tuberculosis control, etc). While the sector-level codes are useful, this high-level categorisation limits one’s understanding of the priorities or scope of projects that make up aid in the health sector.

To disaggregate health sector coded projects further to understand China’s global health priorities, we applied two common frameworks for analysing health aid projects: the OECD CRS purpose code system and IHME’s development assistance for health (DAH) classification structure. The OECD CRS purpose code classification uses five-digit purpose codes that identify the ‘specific areas of the recipient’s economic or social development the transfer intends to foster’.^12^ Purpose codes within the health sector include activities such as malaria control, medical research and family planning. See online supplemental annex 2 for the full list of health aid-related purpose codes in the OECD CRS framework. The IHME database exclusively tracks DAH. IHME’s classification system categorises health aid by health focus or programme area.^13^ See online supplemental annex 3 for an overview of IHME’s classification system. Due to limited project descriptions in the AidData database, we only coded projects according to IHME’s highest level of categorisation (eg, HIV, malaria) and not according to its more disaggregated programme area fields (eg, HIV treatment, HIV care and support). However, we were able to code projects at a more disaggregated level for ‘health system strengthening and sector-wide approaches’. For example, a substantial number of projects, such as those related to medical teams, fall under ‘human resources for health’ while some infrastructure projects fall under ‘other health systems strengthening’. Distinguishing between these two subcategories is useful due to the wide scope of the category as a whole.

We have opted to analyse all health projects using both frameworks for several reasons. First, IHME is broader in nature, with only 10 focus areas, while OECD has 23 narrower categories. Second, these two frameworks are the most common ways to classify and report health aid and we wanted our findings to be comparable with accepted standards and reporting. While these two categorisation systems differ in what types of focus areas are tracked, they each use a mutually exclusive coding system, meaning that a project cannot be considered in more than one category.

### Coding process

For quality control, all coded projects were independently reviewed by two team members. We conducted a pilot test to ensure the coding methodology for relevant health subsectors/focus areas (ie, the IHME and OECD frameworks) could be consistently applied. Ten per cent of all projects eligible for inclusion were part of the pilot process. Once the team was satisfied that (i) all pilot coded projects were coded appropriately and (ii) the codebook reflected all of our underlying assumptions, all remaining projects were then coded. If a project’s description was unclear, the coder would visit the additional sources cited in the project description when available. While most projects in AidData have short descriptions with active hyperlinks to their underlying sources, the level of detail available for projects can be inconsistent.

To code a project, we first read the project description noting any keywords such as “hospital”, “staffing”, “equipment”, “malaria”, etc. These keywords, along with overall descriptions, provide context on the project’s purpose. After analysing the project description, each project was assigned to its most relevant OECD and IMHE code. Although a project may focus on many dimensions of the health system, each project can only be assigned to one category within each framework (ie, categories are mutually exclusive). According to OECD guidelines, ‘within each sector, care should be taken to allocate supplies, equipment and infrastructure to the most specific code available’.^12^ Therefore, each project should be coded based on the project’s *primar*y focus. For example, if a project is related to building hospitals for malaria, this project would be categorised under the CRS classification as ‘malaria control’ rather than ‘medical services’ or ‘basic health infrastructure’ since malaria control is the primary purpose of the project. See online supplemental annex 4 for a sample project entry that would be categorised under malaria control (OECD CRS) and malaria (IHME).

There are a few OECD CRS codes that are fairly similar in nature and require additional nuance to determine the most appropriate code. Therefore, we developed a clear approach for navigating these types of projects to ensure consistency in our coding methods. For instance, basic health care focuses on basic primary healthcare programmes, where activities are focused on achieving universal health coverage, such as routine vaccines. Medical services focus on more specialised activities and treatment such as funding of laboratories, equipment for specialised surgeries or ambulances would be considered under this category. Details on our approach, assumptions and resources used to make such determinations can be found in online supplemental annex 5.

### Estimating missing financial values

The AidData dataset lacks financial values for many health projects in the database: of the 1339 projects included in our analysis, only 541 projects (40.5%) had an assigned financial value. We did not know if these data were missing at random. To estimate missing financial values, we took several approaches.

First, we calculated the median and average values for projects that had financial data according to a project’s subsector and flow type. Average values are likely to overestimate financial resources for a project given the skewed nature of the available data. Ultimately, we opted to use median project values in our analysis since this is the most conservative approach. More details on our approach to using median values is outlined below.

The median value for projects was determined by two key factors: subsector (eg, malaria control) and flow type (eg, grant). Our rationale for choosing these two dimensions is that a malaria grant is more likely to be similar to another malaria grant than it is to be similar to something such as a loan for a tertiary hospital. We identified median values based on available financial data according to the subsectors from each framework and came up with very similar results.

Occasionally, there were no financial data available for a particular flow type. In this circumstance, we used the median value for a similar flow type within the same subsector (eg, median value for a malaria grant in place of a free-standing technical assistance project for malaria). If no financial data were available for an entire subsector, we used the median value for that particular flow type agnostic of subsector (eg, if no health personnel projects had any financial data, the median value for each project’s flow type was used). Overall, 63 projects required this type of correction under the OECD framework while 47 projects for the IHME framework required this correction.

We then conducted a regression analysis. The advantage of using this method is that we could predict values for missing projects based on several key factors that could affect a project’s value, such as year disbursed, recipient region, subsector and flow type. However, there are several factors that might reduce the reliability of regression models including the skewed distribution of the available financial data, using a relatively small dataset to predict a larger one, and the limited number of control variables. Although we did not ultimately use this in the main body of our paper, we provided details on the output of this analysis in online supplemental annex 6.

### Comparing China’s health aid with aid from other bilateral donors

To compare China’s health aid against aid from other bilateral donors, we used data from the OECD CRS database, and therefore only rely on the OECD subsector coding framework for this comparison. AidData’s database does not account for disbursements by bilateral donors to multilateral funds and therefore our estimate and comparisons reflected bilateral aid directly to countries only. ODA bilateral disbursements to countries for sector codes ‘health’ (120) or ‘population policies/programmes and reproductive health’ (130) were summed to ensure no multilateral support was included. Disbursement data are only available after 2002 and therefore our cross-donor comparisons spanned a shorter time horizon (2002–2017) than the rest of the analysis.

All financial values were shown in millions of constant 2017 US$. For financial data related to donors other than China, we downloaded data in current prices and converted to constant 2017 US$ using the same method as the AidData database uses, that is, the OECD DAC deflator.^10 14^

## Results

We present our findings in three parts. First, we present an analysis of China’s health aid portfolio based on project counts to show areas of focus and priority within China’s aid portfolio. Second, we supplement our project count analysis with an estimate of China’s health aid portfolio from a financial standpoint, using median values for projects with missing financial data. Finally, we compare China’s health aid portfolio with that of other donors.

### China’s health aid portfolio by project count

**Figure 2:**
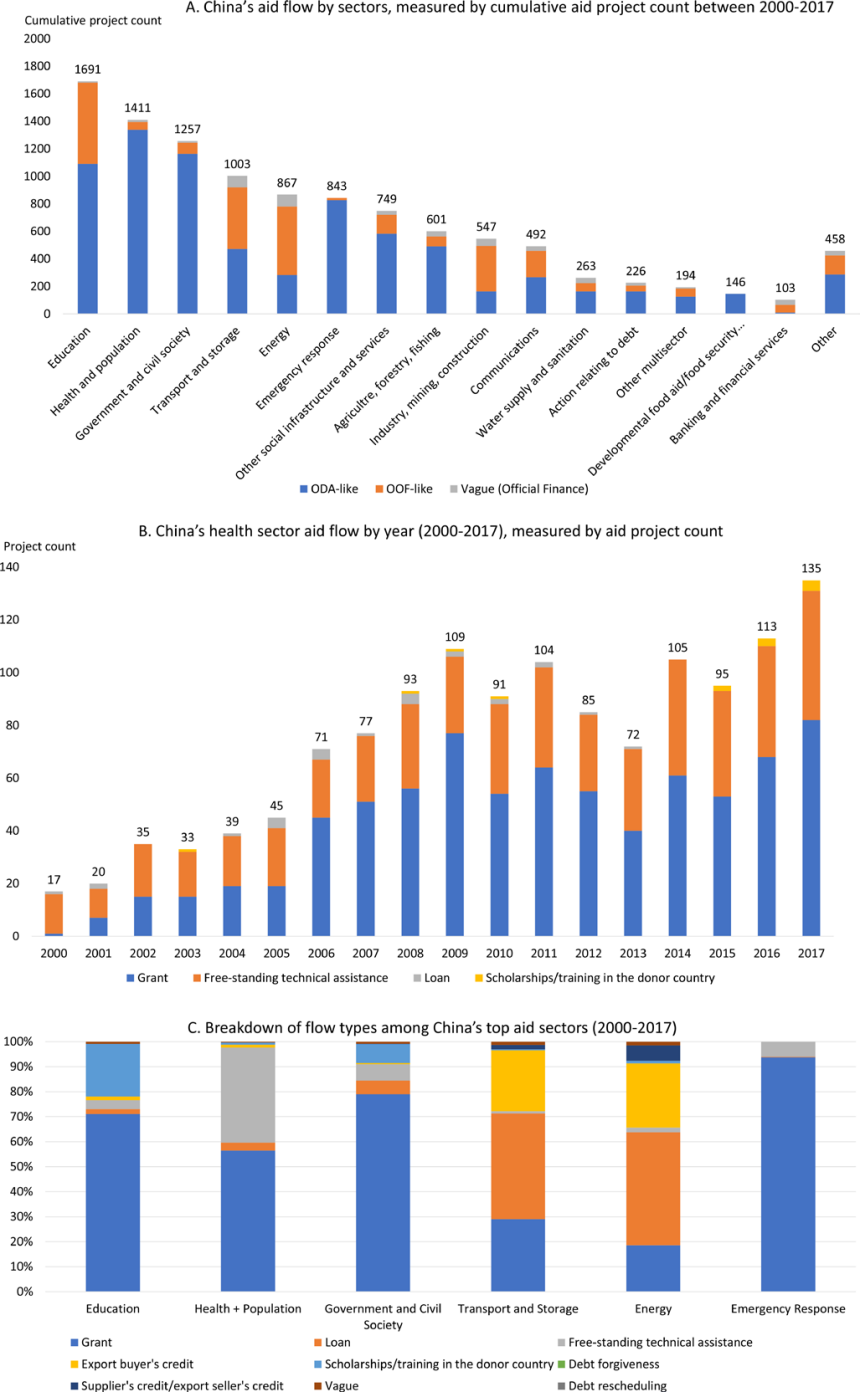
China’s aid flow. (A) China’s aid flow by sectors, measured by cumulative aid project count between 2000 and 2017; (B) China’s health sector aid flow by year (2000–2017), measured by aid project count; (C) breakdown of flow types among China’s top aid sectors (2000–2017). Include all official finance. From left to right, ranked by project count with education being the largest sector and emergency response being smallest sector. ODA, official development assistance. OOF, other official flows.

Over the period 2000–2017, the health sector was the second largest sector in China’s official development financing portfolio, behind the education sector (figure 2A). However, a key difference between the two sectors is the nature of flows: projects in the health sector were predominately aid-based (95% of projects were ODA-like in nature) whereas projects in the education sector were only 64% ODA-like, meaning other forms of official flows played a larger role in this sector.

The volume of health ODA-like projects increased over time from 2000 to 2017 (figure 2B). In particular, there was a substantial uptick in projects beginning in 2006. Over 85% of all health aid projects occurred in 2006 or later. These projects were overwhelmingly in the form of grants (58%) and technical assistance (39%). Although loans generally have a higher financial value than grants or technical assistance, they made up a very small portion of the overall project portfolio (2% of total projects). Scholarships for study in China made up 1% of total projects. The large role that free-standing technical assistance plays within the health sector (n=537, 47%, figure 2C) is not seen with other sectors: among China’s top five sectors pictured in figure 2C, free-standing technical assistance plays a much smaller role (ranging between 1% and 7%). Almost all health aid projects (n=1310, 98%) were funded by Chinese government agencies, while the remaining projects were funded by state-owned companies (n=21, 1.4%) and state-owned policy banks (n=8, 0.5%).

**Figure 3:**
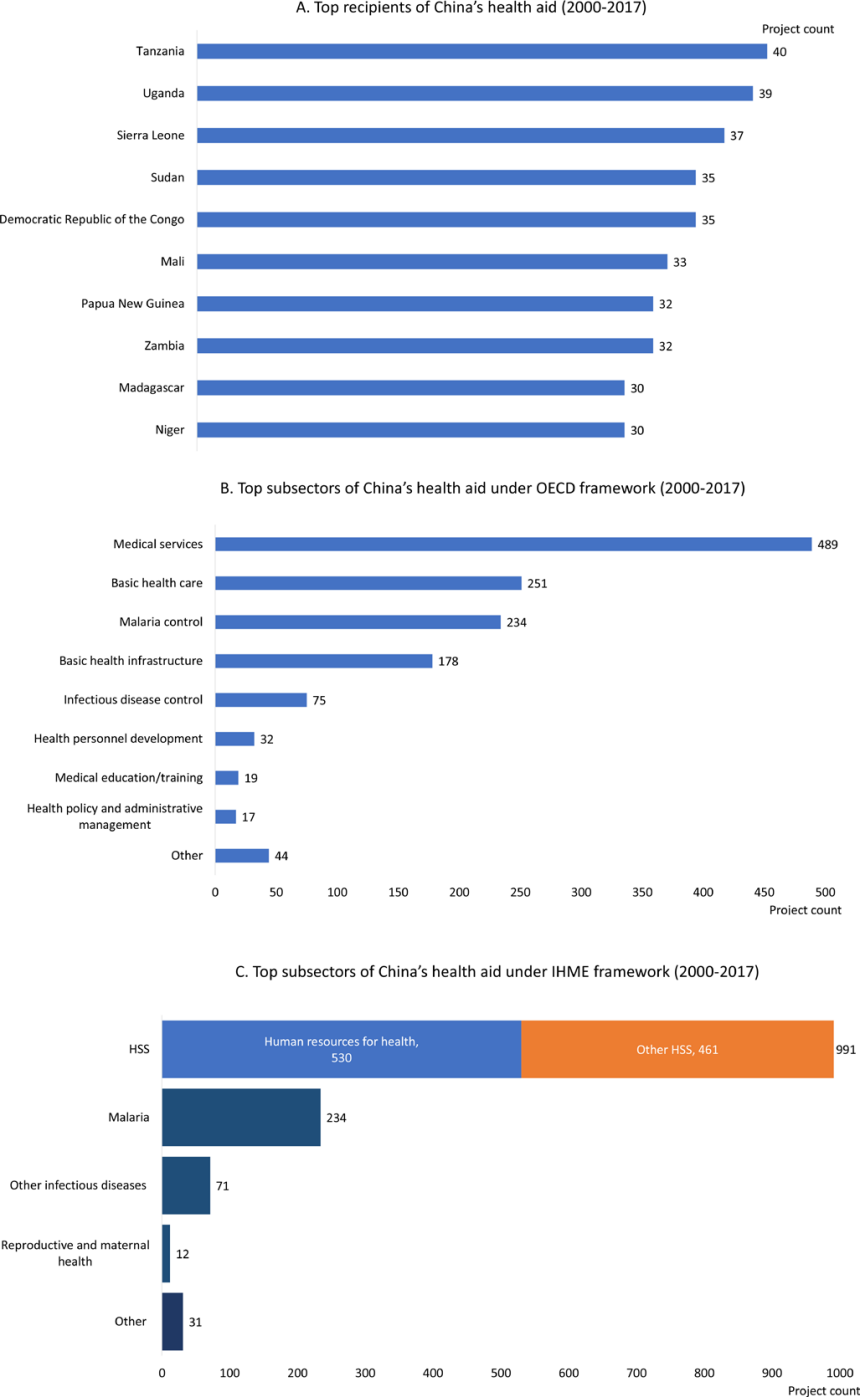
China’s health aid priorities, measured by cumulative aid project count between 2000 and 2017. (A) Top recipients of China’s health aid (2000–2017); (B) top subsectors of China’s health aid under OECD framework (2000–2017); (C) top subsectors of China’s health aid under IHME framework (2000–2017). HSS, health system strengthening; IHME, Institute for Health Metrics and Evaluation; OECD, Organisation for Economic Co-operation and Development.

All global regions received at least one Chinese health aid project. Countries in Africa received most of these projects (75%), followed by Asia (10%) and the Pacific (6%). The annual number of health aid projects to Africa increased from 2006. Between 2000 and 2006, the average number of annual health aid projects to Africa was 24. The average annual project count rose to 73 between the period of 2007–2017. Among the top health aid recipients, only one country lies outside of sub-Saharan Africa (Papua New Guinea) (figure 3A).

There are very clear areas of focus for China’s health aid portfolio. Applying the OECD CRS framework, we find that over 90% of all projects fell into just five sectors: medical services, basic health care, malaria control, basic health infrastructure and infectious disease control (figure 3B). These priority areas have shifted over time. While malaria was the top subsector in the mid-2000s, its importance to China’s profile has declined in recent years (online supplemental annex 7 figure A1). In recent years, medical services and basic health care have continued to make up the majority of China’s portfolio (40% and 22% of annual projects in 2017, respectively).

Looking at health focus areas from the IHME framework shows an even more concentrated area of focus. Three subsectors alone made up 97% of all health aid: health system strengthening (HSS, 74%), malaria (18%) and other infectious diseases (5%) (figure 3C and online supplemental annex 7 figure A2). HSS primarily focused on human resources for health via Chinese medical teams and other cross-disease areas of investment, such as hospital or clinic infrastructure. HSS was the top subsector for each region. In Africa and the Pacific, HSS focused primarily on human resources for health while in Asia, the Americas, Europe and the Middle East, HSS projects were predominately other types, such as infrastructure.

### Financial estimates of China’s health aid portfolio

**Figure 4:**
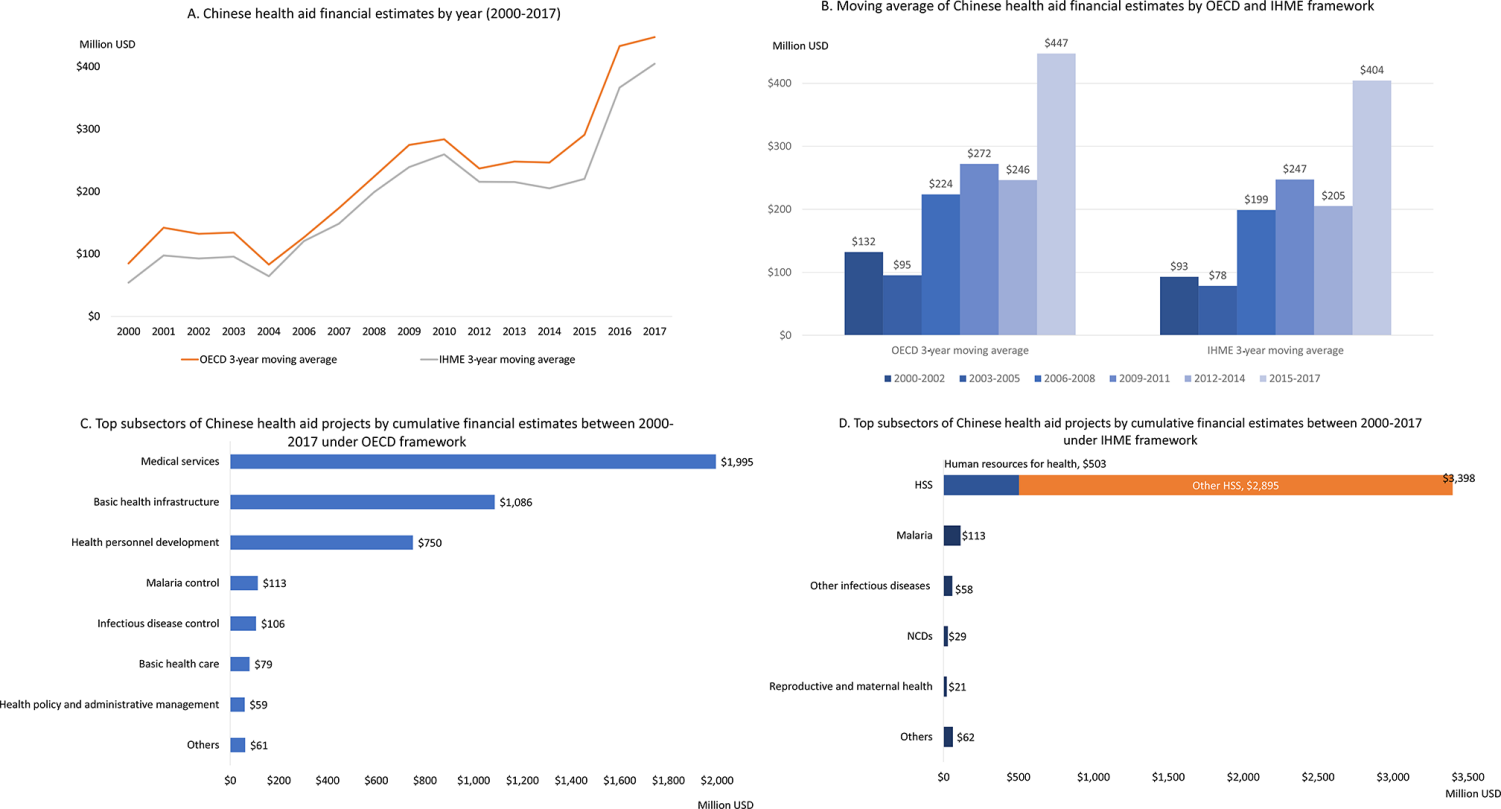
Chinese health aid financial estimates. (A) Chinese health aid financial estimates by year (2000–2017); (B) moving average of Chinese health aid financial estimates by OECD and IHME framework; (C) top subsectors of Chinese health aid projects by cumulative financial estimates between 2000 and 2017 under OECD framework; (D) top subsectors of Chinese health aid projects by cumulative financial estimates between 2000 and 2017 under IHME framework. HSS, health system strengthening; IHME, Institute for Health Metrics and Evaluation; OECD, Organisation for Economic Co-operation and Development; NCDs, non-communicable diseases.

The cumulative total of Chinese health aid reached about US$4 billion over the 18-year period of 2000–2017: this figure was slightly higher using the medians for the OECD framework (US$4.25 billion) than for the IHME framework (US$3.68 billion). As mentioned, annual amounts of Chinese health aid have shown an upward trend since 2000, although there have been fluctuations upwards and downwards between years (figure 4A). Figure 4B shows a 3-year moving average of both OECD and IHME estimates (left) and a breakdown of the median values according to each estimation method (right).

Financial estimates using the OECD framework show concentration in three key subsectors (figure 4C). The top three sectors contributed to 90% of total aid from 2000 to 2017: medical services (47%), basic health infrastructure (26%) and health personnel development (18%). These subsectors are different from the top subsectors ranked by total project counts. For example, basic health care received the highest number of projects yet it is ranked sixth using financial estimates. Meanwhile, there were fewer infrastructure-related projects than basic health care projects from 2000 to 2017, but infrastructure-related projects were costlier per project.

Financial estimates using the IHME framework reinforce that HSS is the primary subsector for health aid, making up 92% of the total (figure 4D). However, despite making up fewer projects, infrastructure under the ‘other HSS’ category makes up 85% of the total financial estimate, likely due to health-related infrastructure projects being included in this category. Malaria was also a priority area, but made up only 3% of total health aid.

**Figure 5:**
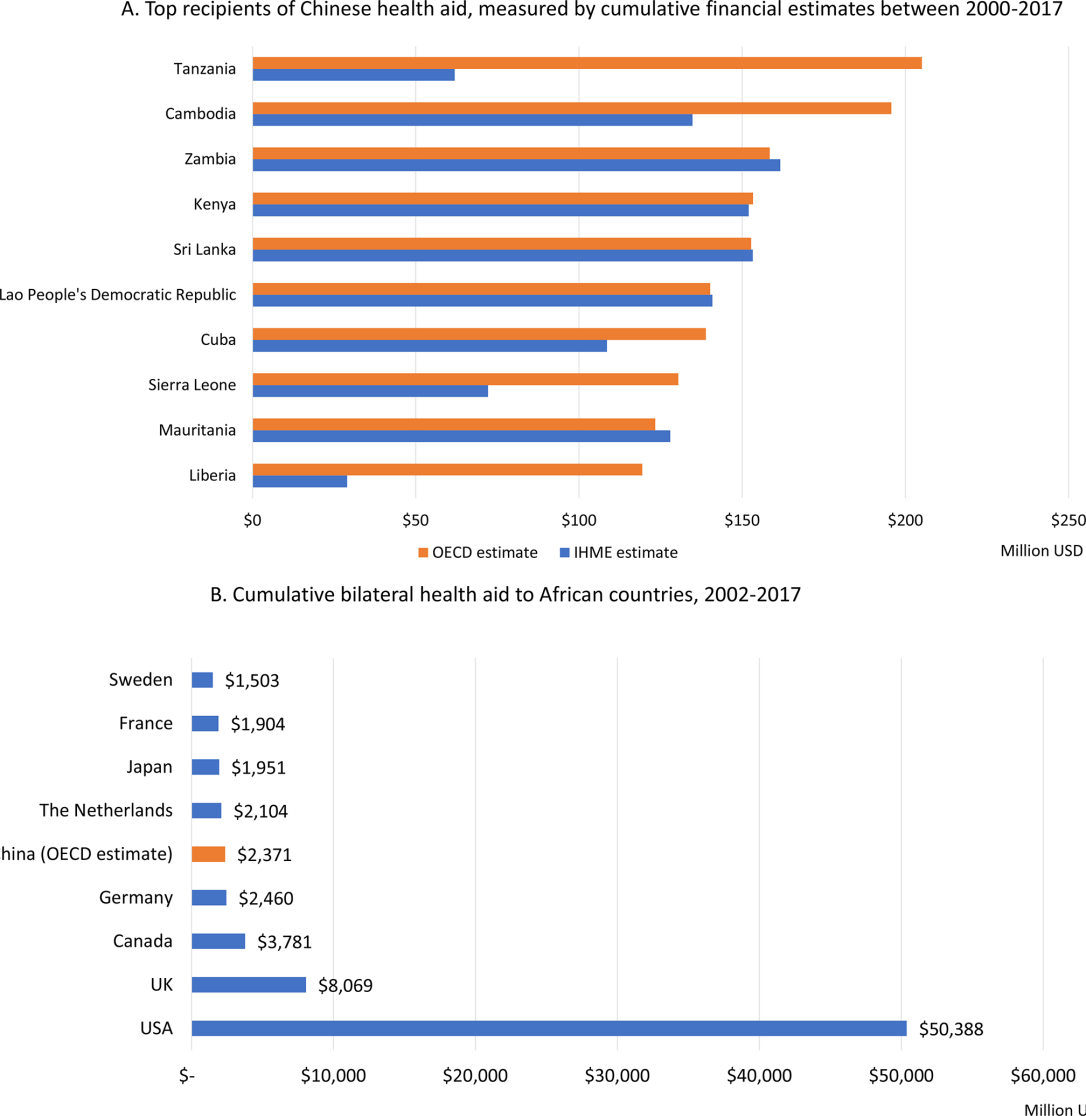
(A) Top recipients of Chinese health aid, measured by cumulative financial estimates between 2000 and 2017; (B) cumulative bilateral health aid to African countries, 2002–2017. Values shows in millions, US$ constant 2017. China estimate is based on OECD framework (US$2371). The IHME framework estimate is US$2078. In total, there are 23 Developmental Assistance Committee (DAC) members. Membership can be found here: https://www.oecd.org/dac/development-assistance-committee/. AidData’s database does not account for disbursements to multilateral funds and therefore, our estimate and comparisons reflect bilateral aid to countries only. IHME, Institute for Health Metrics and Evaluation; OECD, Organisation for Economic Co-operation and Development.

Africa received the most financial health aid of any region (60% of the total), followed by Asia (22%). The top 10 recipient countries are located in Africa and Asia (figure 5A).

### Financial comparison with bilateral health aid from DAC donors

From 2002 to 2017, China’s cumulative global financial health aid contributions were comparable to DAC donors such as Australia, Norway and Sweden. However, given China’s strong geographic focus in Africa, if we restrict our comparison to bilateral health aid in Africa only, the picture changes slightly. Compared with DAC donors, China was the fifth largest health aid donor in Africa from 2002 to 2017 under the OECD framework, and the sixth largest under the IHME framework (figure 5B). If we further focus on the comparison for only the most recent 10 years, China was the fifth largest health aid donor in Africa from 2008 to 2017. The USA is by far the largest bilateral health donor in African countries, followed by the UK. However, China had very similar levels of health aid to Germany, the Netherlands and Japan. Similarly, most donors delivered the bulk of their health ODA in the form of grants, although some donors did provide a portion of their health portfolios via loans (eg, 19% of France’s health ODA, 17% of Germany’s ODA and 14% of Japan’s ODA).

Compared with China’s top OECD subsectors (medical services, basic health infrastructure, health personnel development, malaria control, basic health care, infectious disease control), top health aid DAC donors focus on different priorities (online supplemental annex 7 figure A3). Across all DAC donors, STD control including HIV/AIDS was the largest subsector of focus (42% of all DAC health ODA), followed by basic health care (9%), infectious disease control (8%) and reproductive healthcare (8%). Infectious diseases, including HIV/AIDS and/or reproductive healthcare more broadly, were clear priority areas for each of the leading donors. Medical services, China’s top subsector, was only among the top five subsectors for Japan (15% of health aid portfolio) and France (7% of health aid portfolio).

## Discussion

This study aims to build on previous tracking efforts and create a more disaggregated and standardised account of China’s global health aid footprint. Our analysis found that health is a major focus area of China’s aid portfolio, making up the largest share of ODA-like projects out of all sectors, and the second largest share of projects behind the education sector when all types of official development finance are considered.

Annual levels of Chinese health aid increased over time, from 2000 to 2017, with a particularly sharp uptick after 2006. This increase was primarily due to China’s health aid to Africa, which makes up most of its support. This increased focus on health in Africa coincided with China’s first white paper on ‘China’s African Policy’, which highlighted China’s intention to increase its support to the region, including health-related support.^15^ Specifically, this white paper, in addition to China’s most recent foreign aid white paper, highlighted China’s intention to continue sending medical teams, provide medicines and equipment, train medical personnel, assist with treatment and control of infectious diseases and train personnel on how to use traditional Chinese medicines.^4 15^ The Forum on China-Africa Cooperation (FOCAC), established in 2000, is another regularly occurring platform where China pledges its support and highlights its intended health assistance for the succeeding years.^16^ Health aid has played a major role in previous FOCAC summits and continues to do so today.^16^

We used two different methods for assessing health subsectors and focus areas: the OECD CRS framework and the IHME framework (online supplemental annex 2 and 3). Although these two frameworks have important differences, when we used them for our analysis they told similar stories: infrastructure support and medical teams are the primary areas of focus for China’s health aid. These focus areas are distributed across several OECD codes depending on the type of infrastructure project (eg, a primary care facility or a tertiary hospital) or the level of services provided (eg, basic health care or specialised services) (see online supplemental annex 5 for details). However, these same infrastructure and medical team projects are focused within one IHME code: HSS. Infrastructure-related projects are fewer in number, but are more expensive per project, and therefore rank higher when we look at China’s health aid portfolio from a financial point of view than a project count point of view.

Using both frameworks to estimate missing project values gives fairly similar estimates of about US$4 billion in cumulative total Chinese health aid across the 18-year time period we assessed (2000–2017). However, this similarity between the results of using the two different frameworks may be due to the method we used to estimate missing values, using subsector and flow type as our primary inputs. We recognise that this is an imperfect solution and could underestimate or overinflate aid flows. However, we know that missing financial data dramatically underestimates the Chinese health aid footprint and we consider our approach an improvement on the currently available estimates. We also present findings according to project counts as another way to measure Chinese health aid priorities and trends decoupled from financial values.

When compared with other donors, China is among the top 10 global health contributors. Furthermore, given China’s geographic concentration in Africa, when compared with bilateral flows of DAC donors in the region, China emerges as a major health aid provider. China provided similar levels of support to Africa as other top donors of the Millennium Development Goal era, such as Germany, the Netherlands and Japan. However, the subsectors of focus between the portfolios of China and leading DAC donors during this time period diverges. DAC donors had a heavy emphasis on infectious disease control, including HIV/AIDS, and on reproductive health and family planning. China does contribute to these subsectors, although they were not among the top priority subsectors.

Our study has similar findings to the results of other studies of Chinese health aid, which also show that China is undoubtedly becoming an emerging donor in health with increasing health aid commitments^7 17–19^ particularly concentrated in the African region.^19 20^ Within China’s unique health aid portfolio, our study showed that there is a strong focus on HSS and malaria, a finding seen in other studies.^19^ Both our study and a study by Liu *et al* found that the proportion of loans out of China’s total support was lower for health aid compared with the proportion to other sectors.^7^ Our financial estimates of China’s health aid are generally lower than estimates by other studies, mainly because we restrict our analysis to the bilateral health ODA, which excludes multilateral support, and we exclude broader health-related support such as water, sanitation and hygiene.^19 20^ Grépin *et al* used the ‘average’ for missing values while we used the median, which we believe would be less affected by outliers.^19^

A major value of this study is that we restricted the scope to adhere to commonly accepted standards of aid (health ODA, exclusive of allied sectors for health and non-ODA flows) and we classified projects in a systematic way that aligns with other aid tracking efforts (OECD and IHME). To the best of our knowledge, our study also has the longest time span (18 years) of any existing health-specific analyses. It also has a global geographic scope. Nevertheless, our study also has limitations. The lack of financial estimates for over half of China’s health aid projects could potentially lead to overestimate and underestimates on the financial value of China’s health aid. We applied different approaches for financial estimates and also analysed project counts to present a comprehensive picture of China’s health aid footprint. Additionally, AidData does not track disbursements in the same manner as the OECD CRS tracks them, and therefore year on year comparisons are challenging when looking at Chinese aid and DAC donor aid. However, we used the best proxy available for disbursements (ie, only projects that have a formal commitment, are being implemented, or are completed) and we focus our assessment on cumulative aid rather than on a single snapshot in time. Finally, although we used a standardised coding process using accepted frameworks, these codes are mutually exclusive, and therefore could mask some project focus areas that are not the primary purpose of a project (eg, when a medical team provides a range of services across multiple subsectors).

There are several implications for our findings. From a resource mobilisation point of view, databases like the OECD CRS are used to determine current funding levels of DAC donors. Our analysis could add value to these efforts by providing a more complete picture of health aid flows and priorities. Fellow donors would benefit from understanding China’s contributions and priorities for a variety of reasons, such as efforts to increase collaboration, minimise duplicative efforts or determine where there may be funding gaps. From an aid recipient point of view, understanding China’s priority investment areas could help them in a number of ways, such as by improving their bargaining power with other donors or opening the door for new funding for subsectors of their health systems that other donors do not currently prioritise.

## Conclusion

Chinese health aid showed an upward trend from 2000 to 2017, with some fluctuations, and most Chinese health aid projects were in Africa. Our estimate of total cumulative health aid from China between 2000 and 2017, of around US$4 billion, represents an attempt to account for projects in the AidData database that were missing financial values. China is estimated to be the fifth largest health aid donor to African countries from 2002 to 2017, after the USA, the UK, Canada and Germany. These findings enable a better understanding of Chinese health aid in the absence of transparent aid reporting. We believe that such an understanding could lead to better coordination, collaboration and resource allocation for both fellow donors and recipient countries.

## Data Availability

Data are available in a public, open access repository. Data are available from: https://www.aiddata.org/publications/aiddata-tuff-methodology-version-2-0.
