# Supplementary material for: Estimating Chinese bilateral aid for health: an analysis of AidData’s Global Chinese Official Finance Dataset Version 2.0
Source: BMJ Glob Health. 2022 Dec 2;7(12):e010408. doi: 10.1136/bmjgh-2022-010408 (PMC9723905; doi:10.1136/bmjgh-2022-010408)
Supplement: Supplementary data [file bmjgh-2022-010408supp001.pdf]

Estimating Chinese bilateral aid for health: an analysis of AidData's Global Chinese Official Finance Dataset

Annex 1: Projects excluded from analysis after quality control measures

Although these projects fit our inclusion criteria (recommended for research and ODA-like), after careful review, we did not deem that they were appropriately categorized within the health sector. We have provided project ID numbers and a rationale for exclusion in **Table 1**. We excluded these projects from our final counts and estimates.

Table 1: Projects excluded from our analysis

Project ID	Rationale
1484	Recode to humanitarian
2379	Recode to humanitarian
30214	Recode to humanitarian
33231	Recode to humanitarian
34407	Recode to other social infrastructure and services
34816	Recode to humanitarian
35068	Recode to energy
37904	Recode to agriculture
38401	Recode to water, sanitation, hygiene
38575	Recode to humanitarian
52603	Recode to transport and storage
53121	Recode to other social infrastructure and services
55192	Recode to humanitarian
55247	Recode to humanitarian
55813	Recode to other social infrastructure and services
56145	Focused on an individual's care, unlikely to be considered aid
57252	Recode to other social infrastructure and services
57259	Recode to other social infrastructure and services
58920	Recode to other social infrastructure and services
59199	Recode to transport and storage
60723	Recode to humanitarian
60897	Recode to education
62725	Recode to other social infrastructure and services
64153	Recode to agriculture
64445	Recode to other social infrastructure and services

64590	Recode to other social infrastructure and services
64667	Recode to humanitarian
65243	Recode to other social infrastructure and services
65270	Recode to other social infrastructure and services
66628	Recode to agriculture
66778	Recode to other social infrastructure and services
66922	Recode to humanitarian
67615	Recode to other social infrastructure and services
68458	Recode to water, sanitation, hygiene
72126	Recode to industry
72959	Recode to agriculture
73700	Recode to humanitarian
73778	Recode to humanitarian
73779	Recode to humanitarian
73780	Recode to humanitarian
73781	Recode to humanitarian
73784	Recode to humanitarian
73810	Recode to humanitarian
73893	Recode to humanitarian

Annex 2: OECD coding framework

The OECD tracks ODA, which is defined as "government aid designed to promote the economic development and welfare of developing countries".[20] ODA includes grants, "soft loans", and technical assistance.

The OECD classification system assigns each aid project to its most relevant sector, such as ‘health’. Within each sector, more specific classifications, called ‘purpose codes’, are assigned to a project. Each purpose code is mutually exclusive. A list of the purpose codes that are included in this study’s definition of ‘health aid’ (i.e., those categorized as either 'health' or 'population policies/programmes & reproductive health') is shown below (Table 2).

Table 2: OECD CRS purpose code classification system

DAC 5 CODE	CRS CODE	voluntary code	DESCRIPTION	Clarifications / Additional notes on coverage
**120**			**Health**	
**121**			**Health, General**	
	12110		Health policy and administrative management	Health sector policy, planning and programmes; aid to health ministries, public health administration; institution capacity building and advice; medical insurance programmes; including health system strengthening and health governance; unspecified health activities.
		12196	*Health statistics and data*	*Collection, production, management and dissemination of statistics and data related to health. Includes health surveys, establishment of health databases, data collection on epidemics, etc.*
	12181		Medical education/training	Medical education and training for tertiary level services.
	12182		Medical research	General medical research (excluding basic health research and research for prevention and control of NCDs (12382)).
	12191		Medical services	Laboratories, specialised clinics and hospitals (including equipment and supplies); ambulances; dental services; medical rehabilitation. Excludes noncommunicable diseases (123xx).
**122**			**Basic Health**	
	12220		Basic health care	Basic and primary health care programmes; paramedical and nursing care programmes; supply of drugs, medicines and vaccines related to basic health care; activities aimed at achieving universal health coverage.
	12230		Basic health infrastructure	District-level hospitals, clinics and dispensaries and related medical equipment; excluding specialised hospitals and clinics (12191).
	12240		Basic nutrition	Micronutrient deficiency identification and supplementation; Infant and young child feeding promotion including exclusive breastfeeding; Non-emergency management of acute malnutrition and other targeted feeding programs (including complementary feeding); Staple food fortification including salt iodization; Nutritional status monitoring and national nutrition surveillance; Research, capacity building, policy development, monitoring and evaluation in support of these interventions. Use code 11250 for school feeding and 43072 for household food security.

	12250		Infectious disease control	Immunisation; prevention and control of infectious and parasite diseases, except malaria (12262), tuberculosis (12263), HIV/AIDS and other STDs (13040). It includes diarrheal diseases, vector-borne diseases (e.g., river blindness and guinea worm), viral diseases, mycosis, helminthiasis, zoonosis, diseases by other bacteria and viruses, pediculosis, etc.
	12261		Health education	Information, education and training of the population for improving health knowledge and practices; public health and awareness campaigns; promotion of improved personal hygiene practices, including use of sanitation facilities and handwashing with soap.
	12262		Malaria control	Prevention and control of malaria.
	12263		Tuberculosis control	Immunisation, prevention and control of tuberculosis.
	12281		Health personnel development	Training of health staff for basic health care services.
**123**			**Non-communicable diseases (NCDs)**	
	12310		NCDs control, general	Programmes for the prevention and control of NCDs which cannot be broken down into the codes below.
	12320		Tobacco use control	Population/individual measures and interventions to reduce all forms of tobacco use in any form. Includes activities related to the implementation of the WHO Framework Convention on Tobacco Control, including specific high-impact demand reduction measures for effective tobacco control.
	12330		Control of harmful use of alcohol and drugs	Prevention and reduction of harmful use of alcohol and psychoactive drugs; development, implementation, monitoring and evaluation of prevention and treatment strategies, programmes and interventions; early identification and management of health conditions caused by use of alcohol and drugs [excluding narcotics traffic control (16063)].
	12340		Promotion of mental health and well-being	Promotion of programmes and interventions which support mental health and well-being resiliency; prevention, care and support to individuals vulnerable to suicide. Excluding treatment of addiction to tobacco, alcohol and drugs (included in codes 12320 and 12330).
	12350		Other prevention and treatment of NCDs	Population/individual measures to reduce exposure to unhealthy diets and physical inactivity and to strengthen capacity for prevention, early detection, treatment and sustained management of NCDs including: Cardiovascular disease control: Prevention, screening and treatment of cardiovascular diseases (including hypertension, hyperlipidaemia, ischaemic heart diseases, stroke, rheumatic heart disease, congenital heart disease, heart failure, etc.). Diabetes control: Prevention, screening, diagnosis, treatment and management of complications from all types of diabetes. Exposure to physical inactivity: Promotion of physical activity through supportive built environment (urban design, transport), sports, health care, schools and community programmes and mass media campaign. Exposure to unhealthy diet: Programmes and interventions that promote healthy diet through reduced consumption of salt, sugar and fats and increased consumption of fruits and vegetables e.g., food reformulation, nutrient labelling, food taxes, marketing restriction on unhealthy foods, nutrition education and counselling, and settings-based interventions (schools, workplaces, villages, communities). Cancer control: Prevention (including immunisation, HPV and HBV), early diagnosis (including pathology), screening, treatment (e.g., radiotherapy, chemotherapy, surgery) and palliative care for all types of cancers. Implementation, maintenance and improvement of cancer registries are also included. Chronic respiratory diseases: Prevention, early diagnosis and treatment of chronic respiratory diseases, including asthma. Excludes: Tobacco use control (12320), Control of harmful use of alcohol and drugs (12330), research for the prevention and control of NCDs (12382).

	12382		Research for prevention and control of NCDs	Research to enhance understanding of NCDs, their risk factors, epidemiology, social determinants and economic impact; translational and implementation research to enhance operationalisation of cost-effective strategies to prevent and control NCDs; surveillance and monitoring of NCD mortality, morbidity, risk factor exposures, and national capacity to prevent and control NCDs.
**130**			**Population Policies/Programmes & Reproductive Health**	
	13010		Population policy and administrative management	Population/development policies; demographic research/analysis; reproductive health research; unspecified population activities. (Use purpose code 15190 for data on migration and refugees. Use code 13096 for census work, vital registration and migration data collection.)
		13096	*Population statistics and data*	*Collection, production, management and dissemination of statistics and data related to Population and Reproductive Health. Includes census work, vital registration, migration data collection, demographic data, etc.*
	13020		Reproductive health care	Promotion of reproductive health; prenatal and postnatal care including delivery; prevention and treatment of infertility; prevention and management of consequences of abortion; safe motherhood activities.
	13030		Family planning	Family planning services including counselling; information, education and communication (IEC) activities; delivery of contraceptives; capacity building and training.
	13040		STD control including HIV/AIDS	All activities related to sexually transmitted diseases and HIV/AIDS control e.g., information, education and communication; testing; prevention; treatment, care.
	13081		Personnel development for population and reproductive health	Education and training of health staff for population and reproductive health care services.

Source: Purpose Codes: sector classification[10]

Annex 3: IHME coding framework

The IHME tracks DAH. The IHME compiles its data from that of the OECD CRS and other sources such as tax filings or financial statements, thus creating a broader estimate of aid than ODA. The IHME disaggregates data into health focus areas (e.g., HIV) and program areas (e.g., treatment). The relevant DAH tracking codes for IHME's framework can be found in **Table 3** below.

While IHME has very detailed program areas, such as HIV prevention, the project descriptions available in AidData's database did not usually have sufficient information to enable coding at this this level. Therefore, we only coded projects to the highest level of the focus area (e.g., HIV), which corresponds to the leftmost column category in the table below. The only category we were able to disaggregate by program area was health systems strengthening.

Table 3: IHME DAH focus areas

Focus area	Variables	Description
HIV	hiv_dah_18<br>hiv_care_dah_18<br>hiv_ct_dah_18<br>hiv_hss_other_dah_18<br>hiv_hss_hrh_dah_18<br>hiv_treat_dah_18<br>hiv_ovc_dah_18<br>hiv_pmtct_dah_18<br>hiv_prev_dah_18<br>hiv_amr_dah_18<br>hiv_other_dah_18	Funds for health disbursed from source to channel to recipient country for HIV/AIDS, disaggregated by care and support, counseling & testing, other health system strengthening, human resources, treatment, orphans & vulnerable children, prevention of mother to child transmission, prevention, drug resistance, and other.
Malaria	mal_dah_18<br>mal_comm_con_dah_18<br>mal_con_nets_dah_18<br>mal_con_irs_dah_18<br>mal_con_oth_dah_18<br>mal_diag_dah_18<br>mal_hss_other_dah_18<br>mal_hss_hrh_dah_18<br>mal_treat_dah_18<br>mal_amr_dah_18<br>mal_other_dah_18	Funds for health disbursed from source to channel to recipient country for malaria, disaggregated by community outreach, bednets, indoor spraying, other control, diagnosis, other health system strengthening, human resources, treatment, drug resistance, and other

Reproductive and maternal health	rmh_dah_18<br>rmh_fp_dah_18<br>rmh_hss_other_dah_18<br>rmh_hss_hrh_dah_18<br>rmh_mh_dah_18<br>rmh_other_dah_18	Funds for health disbursed from source to channel to recipient country for reproductive and maternal health, disaggregated by family planning, other health system strengthening, human resources, other maternal health, and other
Newborn and child health	nch_dah_18<br>nch_cnn_dah_18<br>nch_cnv_dah_18<br>nch_hss_hrh_dah_18<br>nch_hss_hrh_dah_18<br>nch_other_dah_18	Funds for health disbursed from source to channel to recipient country for newborn and child health, disaggregated by nutrition, vaccines, other health system strengthening, human resources, and other
Non-communicable diseases	ncd_dah_18<br>ncd_mental_dah_18<br>ncd_hss_other_dah_18<br>ncd_hss_hrh_dah_18<br>ncd_tobac_dah_18<br>ncd_other_dah_18	Funds for health disbursed from source to channel to recipient country for non-communicable diseases, disaggregated by mental health, other health system strengthening, human resources, tobacco initiatives, and other
Other infectious diseases	oid_dah_18<br>oid_hss_other_dah_18<br>oid_hss_hrh_dah_18<br>oid_ebz_dah_18<br>oid_zika_dah_18<br>oid_amr_dah_18<br>oid_other_dah_18	Funds for health disbursed from source to channel to recipient country for other infectious diseases, disaggregated by other health system strengthening, human resources, Ebola, Zika, antimicrobial resistance, and other
Tuberculosis	tb_dah_18<br>tb_diag_dah_18<br>tb_hss_other_dah_18<br>tb_hss_hrh_dah_18<br>tb_treat_dah_18<br>tab_amr_dah_18<br>tb_other_dah_18	Funds for health disbursed from source to channel to recipient country for tuberculosis, disaggregated by diagnosis, other health system strengthening, human resources, treatment, drug resistance, and other
Health systems strengthening and sector-wide approaches*	swap_hss_total_dah_18<br>swap_hss_hrh_dah_18<br>swap_hss_pp_dah_18<br>swap_hss_other_dah_18	Funds for health disbursed from source to channel to recipient country for health systems strengthening and sector-wide approaches, disaggregated by human resources, pandemic preparedness, and other
Other	other_dah_18	Funds for health distributed from source to channel to recipient country for which we have health focus area information but which is not identified

		as being allocated to any of the other health focus areas listed
Unallocated	unalloc_dah_18	Funds for health disbursed from source to channel to recipient country for which we have no health focus area information

Note: Table adapted from content to be more reader-friendly

Source: IHME DAH Database User Guide (2018)[11]

Annex 4: Coding example

We provide an example of a project included in our analysis (**Table 4**). Based on the information available, this project would be coded as follows: malaria control 12262 (OECD) and malaria (IHME).

Table 4: Coding example

Project ID	Rec. for research	Project title	Project description	Flow class	CRS sector	Status
319	TRUE	Malaria Treatment Center	At the Beijing Summit of the Forum on China-Africa Cooperation (FOCAC) in November 2006, China pledged to construct a malaria prevention and treatment center later designated to be built for Burundi. The opening ceremony was held on March 27, 2008. According to Burundi's Development Assistance Database, China has disbursed 439,811 USD in funding for the center.	ODA-like	Health	Completion

Annex 5: Codebook for projects related to infrastructure and medical teams

Medical teams and infrastructure-related projects are very commonly found in the AidData database. When the primary purpose of these types of projects was focused on a particular disease, it was coded as such. However, identifying the most appropriate code for these types of projects in the absence of a particular disease focus was particularly challenging using the OECD framework since several purpose codes relate to infrastructure and human resources. Compounding this, many projects had fairly limited descriptions or sources available for further investigation. To ensure consistency and transparency in our coding, we developed a codebook to clearly outline when these types of projects should go into particular categories. We have included our rationale and assumptions below.

Coding infrastructure projects under the OECD framework

Infrastructure projects that are not linked to a particular disease focus area have the potential to fall under one of two categories: basic health infrastructure and medical services. Two key pieces of information were used to determine which category was best suited: the size/type of hospital and the type of services/equipment offered.

> **Basic health infrastructure** (purpose code 12230) is defined as "*district-level hospitals, clinics and dispensaries and related medical equipment; excluding specialized hospitals and clinic.*"[10] Infrastructure projects were coded under 'basic health infrastructure' if the project description mentioned district hospitals or fell under the definition of a district-level hospital, using definitions from Disease Control Priorities (see **Table 5**).[21] As needed, we searched the sources provided in the 'sources' column of AidData's database to see if the hospital or equipment in question meets the criteria of a district hospital. In particular, we looked for mentions of things such as number of beds and specialty services available at a given facility.
>
> **Medical services** (purpose code 12191) are defined as "*laboratories, specialized clinics and hospitals (including equipment and supplies); ambulances; dental services; medical*

rehabilitation. Excludes noncommunicable diseases.”[10] If an infrastructure project does not meet the criteria for a district-level hospital and is not linked to a particular disease focus area, it will likely fit the description of medical services. Second-level and third-level hospitals fall under the medical services category because the nature of these hospitals is more specialized (see **Table 5**). A specialized hospital is a hospital admitting primarily patients suffering from a specific disease or affection of one system, or reserved for the diagnosis and treatment of conditions affecting a specific age group or of a long-term nature.[21]

If it was still uncertain which category an infrastructure project should be coded to, then we defaulted to coding the project to basic health infrastructure. We selected this approach after pilot coding several infrastructure projects. We noticed that if specialty services were provided, these services were often referenced in the project description or in the additional sources column. Therefore, if there is no direct mention to some degree of specialization, we coded the infrastructure project to basic health infrastructure.

Table 5: Definitions of levels of hospital care

Terminology and definitions	Alternative terms commonly found in the literature
First-level hospital<br>Few specialties—mainly internal medicine, obstetrics and gynecology, pediatrics, and general surgery<br>Often only one general practice physician or a nonphysician clinician<br>Limited laboratory services available for general analysis but not for specialized pathological analysis<br>50–250 beds	Primary-level hospital<br>District hospital<br>Rural hospital<br>Community hospital<br>General hospital
Second-level hospital<br>More differentiated by function, with as many as 5 to 10 clinical specialties<br>200–800 beds	Regional hospital<br>Provincial (or equivalent administrative area, such as county) hospital<br>General hospital

Terminology and definitions	Alternative terms commonly found in the literature
Third-level hospital<br>Highly specialized staff and technical equipment—for example, cardiology, intensive care unit, and specialized imaging units<br>Clinical services highly differentiated by function<br>Teaching activities in some facilities<br>300–1,500 beds	National hospital<br>Central hospital<br>Academic, teaching, or university hospital

Source: Definitions of Levels of Hospital Care [21]

Coding infrastructure and equipment under the IHME framework

When a project included equipment, infrastructure, and/or medical products, we used the same approach IHME applied in their 2020 paper.[17] Specifically, when these types of projects were *not* specific to a health focus area (e.g., building a hospital versus a malaria treatment center), they were included as 'other HSS'.

Coding medical teams under the OECD framework

Medical team-related projects are very commonly found in AidData's database. If a medical team is provided for a very specific purpose, such as malaria control, then the project should be coded under malaria control. However, in the absence of a specific disease focus, the OECD framework has four potential codes where medical teams could fit: medical education and training (purpose code 12181), health personnel development (purpose code 12281), basic health care (purpose code 12220), or medical services (purpose code 12191). The key distinction that must be made for medical team-related projects is whether or not the medical teams provided education and training to local health care providers or if the medical teams provided services without any training or knowledge transfer components. Once such a distinction has been made, then we coded according to the following methods.

For medical teams with an education/knowledge transfer component

Medical teams that provide some degree of education, training, or knowledge transfer could fit into one of two potential codes: medical education/training (purpose code 12181) or health personnel development (purpose code 12281).

> **Medical education/training** (purpose code 12181) is defined as '*medical education and training for tertiary level services*.'[10]
>
> **Health personnel development** (purpose code 12281) is defined as '*training of health staff for basic health care services*.'[10]

The key difference between these two codes is that one focuses on basic health care (12281) and one focuses on tertiary care (12181). To determine whether or not the focus of education is on tertiary services or basic health care services, we referred to the WHO definitions of primary and tertiary healthcare services.[22] Specifically, the WHO defines these as follows:

1. **Primary services** are usually the first point of contact within a health system and may be provided by general health care workers; they represent a link to more specialized care. Primary services are usually provided locally in a range of settings (typically communities).
2. **Tertiary services** include specialized consultative health care, usually based at national level and provided in hospitals on an inpatient basis.

Essentially if the medical team's training activities fall under primary services, then the project was coded as health personnel development. If the services appear more specialized, then the project was coded under medical education/training. If a project does not appear to have a training component, then it should be categorized under another more relevant code (see next section.)

If the project descriptions were insufficient to make a conclusive decision on whether or not the training was focused on primary or tertiary services, then we defaulted to code the project as health personnel development, which focuses on basic health care services. This code was selected using a similar assumption we made for medical infrastructure

projects: if the projects involved tertiary services, it is likely that these services would be referenced.

For medical teams focused on service delivery only

Some Chinese medical teams provide direct care to recipients in host countries without any training components for local personnel. Therefore, these projects should not be coded under either of the two previously mentioned codes (12181 or 12281). We coded medical teams without training elements under basic health care (purpose code 12220) or medical services (purpose code 12191).

- **Basic health care** (purpose code 12220) is defined as '*basic and primary health care programmes; paramedical and nursing care programmes; supply of drugs, medicines and vaccines related to basic health care; activities aimed at achieving universal health coverage*.'[10]
- **Medical services** (12191) are defined as '*Laboratories, specialised clinics and hospitals (including equipment and supplies); ambulances; dental services; medical rehabilitation. Excludes noncommunicable diseases*.'[10]

For medical teams where there was inadequate information to distinguish between 'medical services' or 'basic health care,' we used 'basic health care' as the default code. If housing was also provided for a medical team, we coded the housing under the same category as we would a medical team since these two items are typically grouped together for other medical team projects.

Coding medical teams under the IHME framework

The IHME framework only has one applicable code for each (under health system strengthening and sector-wide approaches), 'human resources', and 'other'. Therefore, all infrastructure projects with no other primary disease focus are coded under other, and medical team projects

are coded under human resources. Additionally, some projects may involve infrastructure or team components, yet they are coded under different categories because their overall purpose is best suited for another code. For example, if an infrastructure or medical team's primary purpose was to support a particular disease such as malaria, these projects would be considered under malaria codes ('malaria control' or 'mal_dah_18').

Annex 6: Predicting missing values using regression analysis

We conducted multiple linear regression analysis to predict the missing value of projects. We first used projects without missing value to explore the association between financial values of projects with other project information, including year, status, flow, regions and sectors. We operated regression models for OECD CRS category and IHME category separately. Considering the limited sample size, we combined categories with small size. Variables and findings are presented in **Table 6**.

Table 6: Regression analysis

Variables	Model 1 (OECD model)			Model 2 (IHME model)		
	β	Std. Error	P value	β	Std. Error	P value
(Constant)	-420.88	246.14	0.09	-499.03	244.46	0.04
Commitment Year	0.21	0.12	0.08	0.25	0.12	0.04
Status: Implementation or pipeline as reference						
Completion	8.61	1.71	0.00	7.92	1.69	0.00
Flow type: grant as reference						
Free-standing technical assistance	-4.72	2.65	0.08	1.72	4.05	0.67
Loan or scholarships	16.27	2.63	0.00	16.63	2.63	0.00
Recipient region: Africa as reference						
Asia and The Pacific	4.88	1.52	0.00	5.61	1.52	0.00
Oceania	-2.11	2.21	0.34	-2.80	2.21	0.21
America	-0.16	2.04	0.94	0.11	2.03	0.96
Other (Europe, Middle East, Multi-regions)	-3.22	2.35	0.17	-3.63	2.34	0.12
OECD code: Medical services as reference						
Malaria	-6.94	1.56	0.00			
Basic Health	-4.93	2.14	0.02			
Basic Health Infrastructure	-1.45	1.43	0.31			
Infectious Diseases Control	-7.62	1.88	0.00			
All others	-8.00	2.12	0.00			
IHME code: HSS: Human Resources for Health as reference						
HSS: Other HSS				0.04	3.60	0.99
Malaria				7.52	3.25	0.02
Other Infectious Diseases				1.65	3.41	0.63
All others				0.72	4.02	0.86
Funding type						
State	7.76	2.82	0.01	7.19	2.83	0.01

Model parameters: for model 1: R2 = 0.23, F =11.00, P<0.001; for model 2: R2 = 0.22, F =11.56, P<0.001.

Using the findings from the regression models, we predicted the missing values of financial value for the rest of the projects. The financial estimates of Chinese health aids totals to 4.9 billion under OECD framework and 4.3 billion under IHME framework.

The results were significantly higher than the findings we presented in the main report for the following reasons: due to a small sample size and limited variables included in the models, the R squares were relatively low for both models, indicating they were only able to captured small proportion of the variations; second, comparing to applying average or median number for missed values, some of the predicted value from this approach were higher than any single project. We decided to report financial estimates based on median for that approach is easier for audience to follow.

Annex 7: Additional figures

A1. Top sub-sectors of China's health aid under OECD framework, by year (2000-2017)

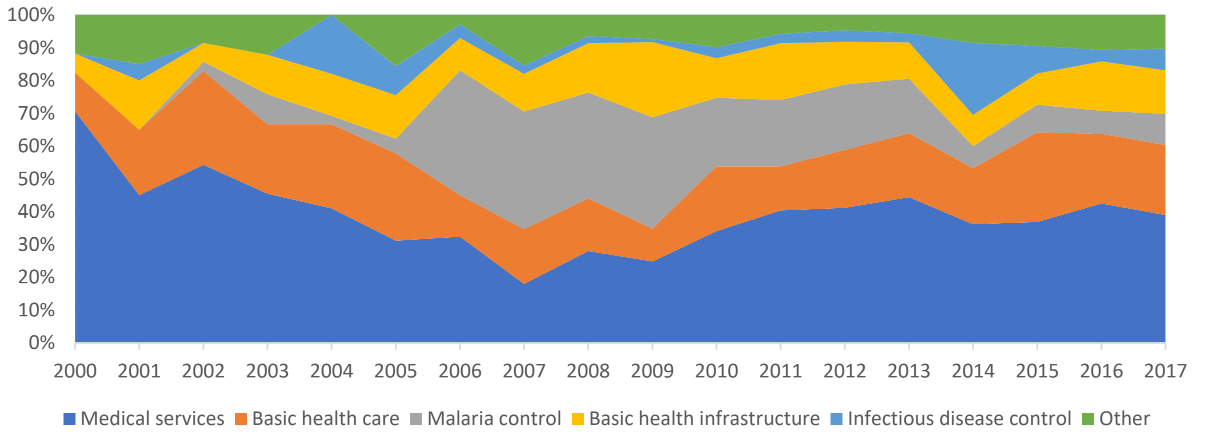

A2. Top sub-sectors of China's health aid under IHME framework, by year (2000-2017)

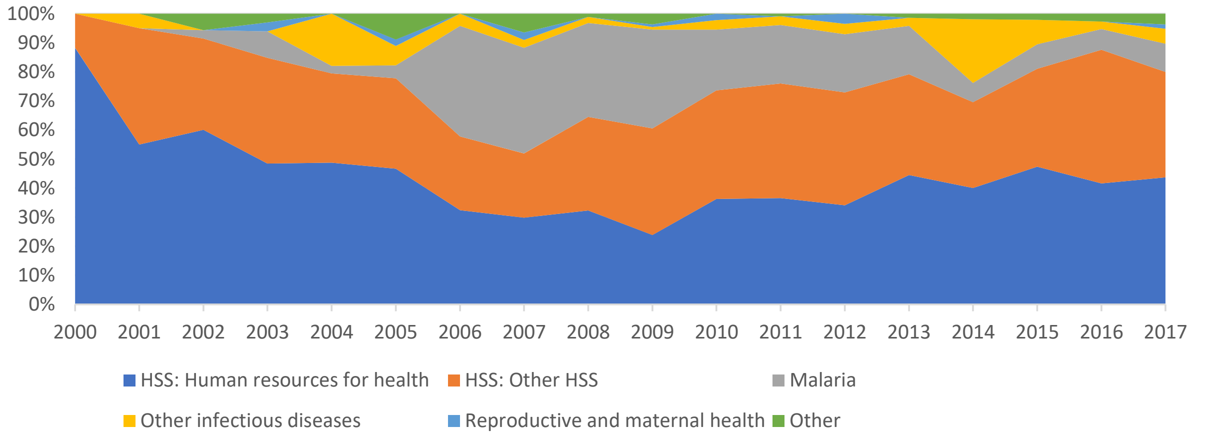

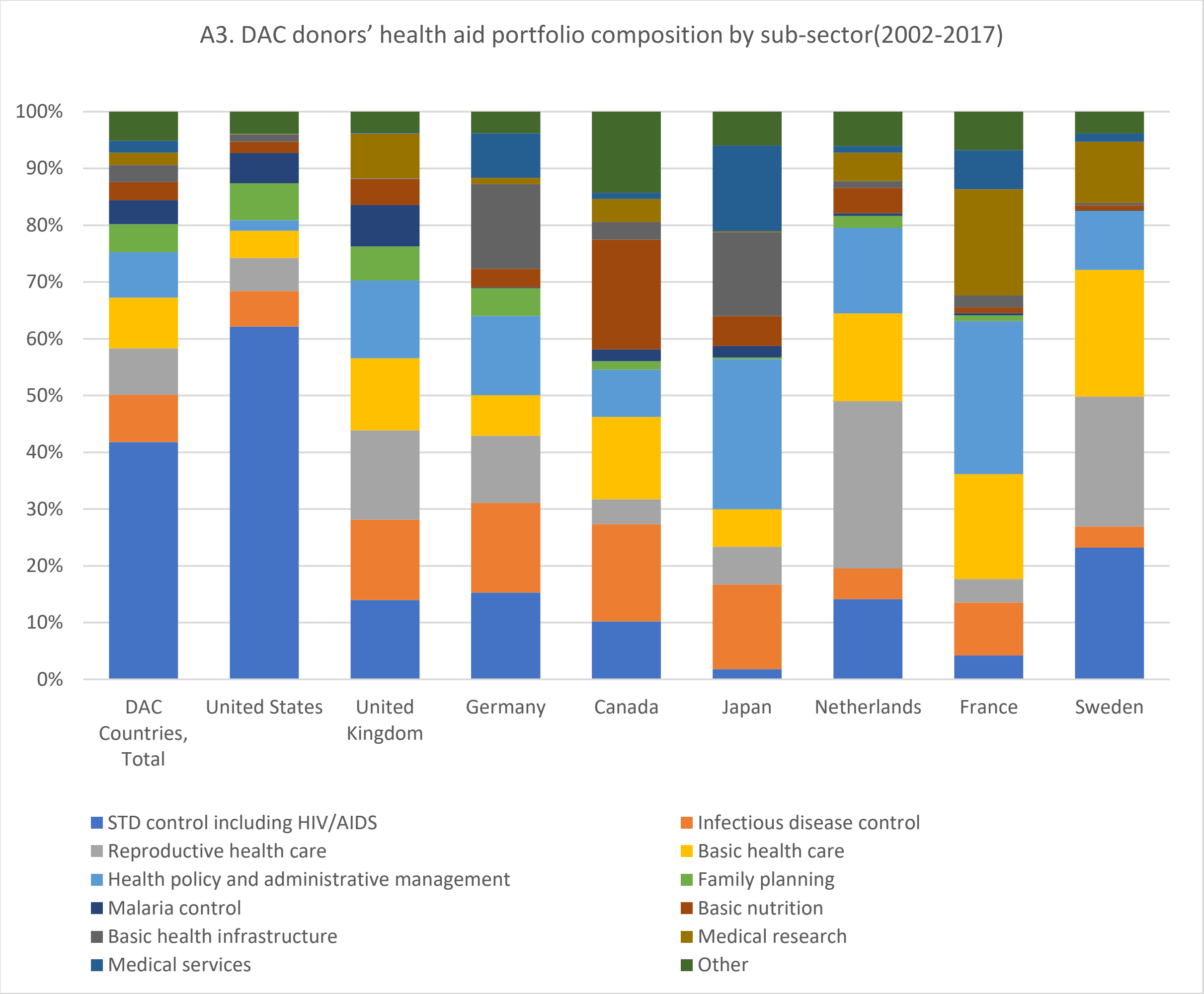
A3. DAC donors' health aid portfolio composition by sub-sector(2002-2017)
100%
90%
80%
70%
60%
50%
40%
30%
20%
10%
0%
DAC Countries, Total
United States
United Kingdom
Germany
Canada
Japan
Netherlands
France
Sweden
STD control including HIV/AIDS
Infectious disease control
Reproductive health care
Basic health care
Health policy and administrative management
Family planning
Malaria control
Basic nutrition
Basic health infrastructure
Medical research
Medical services
Other
